# The effects of restrictive measures due to the COVID-19 pandemic on the extensive farming system of small ruminants

**DOI:** 10.5194/aab-65-157-2022

**Published:** 2022-05-02

**Authors:** Maria D. Yiakoulaki, Eleni T. Tsiobani, Christina-Ioanna G. Galliou, Konstantinos G. Papaspyropoulos

**Affiliations:** 1 Department of Forestry and Natural Environment, Faculty of Agriculture, Forestry and Natural Environment, Aristotle University of Thessaloniki, Thessaloniki, 54124, Greece; 2 Independent researcher

## Abstract

Vigorous restrictive measures were imposed worldwide to control the spread of the COVID-19 pandemic. However, the effects those measures had on livestock production systems have not yet been sufficiently clarified. The literature has focused mainly on the impact of COVID-19 on the intensive livestock production system, especially the meat and milk supply chain, as well as the welfare of animals, highlighting its fragility, but failed to address the way the restrictive measures affected the extensive farming system of small ruminants. In this study, we aimed to investigate the effect of the restrictive measures during the first wave of the COVID-19 pandemic on the parameters of sheep and goat extensive farming systems. We used a survey and personal interviews to record the breeders' profile and the structural characteristics of the holdings, and we showed that the restrictive measures had no effect on the parameters of small ruminant extensive farming systems, except for the daily work routine of breeders, which was negatively affected in holdings with a high number of sheep. We expect this study to be helpful to policy makers regarding formulating interventions for the resilience and the stability of the sheep and goat extensive farming system in future situations of emergency.

## 1 Introduction

COVID-19 emerged from a new variant of coronavirus, SARS-CoV-2, towards the end of 2019 (FAO, 2020a; Zhang, 2020). This virus caused a worldwide unprecedented health and socioeconomic crisis (Malik et al., 2020; Rodriguez-Morales et al., 2020). In February 2020, the World Health Organization classified the virus into a high-risk category, and in March that year, it was declared a pandemic (Tiwari et al., 2020).

The ease of coronavirus transmission led many countries around the world to take restrictive measures to control its spread (Rothan and Byrareddy, 2020). Some of these measures were quarantines, social distancing, travel bans, border controls and restrictions on the import–export of goods, suspension of many businesses and activities, and lockdowns of entire areas (FAO, 2020a). These measures have negatively affected all sectors of the modern economy, including the livestock husbandry sector (FAO, 2020b; Hashem et al., 2020; Grandin, 2021).

Even though the time period is short to estimate the impact of the COVID-19 pandemic on the livestock husbandry sector, it has been reported (Ani et al., 2020; Biswal et al., 2020; Marchant-Forde and Boyle, 2020; Taylor et al., 2020) that the restrictions on animal movements in pastures as well as the disruptions in feedstuff supply have negatively affected livestock farms in several countries (Biswal et al., 2020; FAOb, 2020). The limited access to veterinary services and medication restricted the monitoring of animal health, resulting in a negative impact on their welfare. In addition, this posed a risk for a new pandemic outbreak of animal diseases transmittable to humans (Hashem et al., 2020). Moreover, the shortage of laborers and workers due to lockdowns, border controls, or isolation to quarantines intensified these problems. At the same time, due to the decreased purchasing power, basic priorities, and safety awareness, consumer preferences shifted towards fruits, vegetables, and flour, thus affecting the consumption of dairy and meat products. These changes have in turn affected the resilience and sustainability of livestock enterprises (Wang et al., 2020; Marchant-Forde and Boyle, 2020; Ijaz et al., 2021).

Sheep and goat farming is one of the most important sectors in Greece with a special contribution to the national economy as it accounts for about 18 % of the total agricultural income (Hellenic Ministry of Rural Development and Food, 2011). Greece is the first country in Europe in terms of numbers of farmed goats (3.6 million) and the fourth in terms of farmed sheep (8.4 million) (Hellenic Statistical Authority, 2019). In terms of farming, 85 % of the total number of sheep and 82 % of the total number of goats are reared with the utilization of natural resources in mountainous and disadvantaged areas (Papanastasis, 2009). The majority of these areas (75 %) are state-owned and grazed communally by the livestock of the local people (Yiakoulaki and Papanastasis, 2014). Hence, each farmer who owns livestock can freely utilize the pastures of the village where he resides without any control or management plan. This exploitation practice of Greek pastures has been taking place since the Byzantine Empire times and is considered the main reason for the degradation and reduced productivity of these areas (Papanastasis, 1981, 2009). Recently, the rational use and sustainable management of grazing lands were given significant importance in the national legislation (Common Ministerial Decision 1058/71977/FEK 2331/7.07.2017). However, the application of the legislation is still pending.

The first COVID-19 case appeared in Greece on 26 February 2020, and as the cases increased, rigorous public health measures were imposed in the country from the beginning of March, including a strict nationwide lockdown on 23 March 2020 to control the spread of the pandemic (National Public Health Organization, 2020). These measures included social distancing and restrictions on citizen mobility, suspension of educational, cultural, and economic activities (excluding banks, food stores, and drugstores), quarantines, and the introduction of teleworking in public services and the private sector. During these measures, citizens who could not stay home and had to travel across the country were required to have their police ID or passport with them, as well as a signed certificate stating the purpose of their departure and the length of time they would stay out of their home. Primary agricultural and livestock producers, employees, and self-employed professionals could move to and from work with a permit document that listed their name, home address, work address, and working hours including the estimated arrival and departure time (European Union Agency for Fundamental Rights, 2020). The restrictions were gradually lifted from 4 May 2020 after the 42 d general lockdown. The majority of the population complied with the restrictive measures, and the Greek authorities succeeded in containing the virus during the first wave of the COVID-19 outbreak. However, the pandemic had an economic, social, and political impact on the country (Kousi et al., 2021). In addition, the coronavirus pandemic, which was caused by a novel and unknown infectious disease, made Greek citizens fear for their health and their lives, creating uncertainty for their future, and increased their anxiety (Parlapani et al., 2020).

Research concerning the effect of measures imposed to control the spread of the coronavirus pandemic on the livestock husbandry sector is limited to intensive production systems (Hashem et al., 2020; Defo Deeh et al., 2020; Marchant-Forde and Boyle, 2020), and there are no data about their effect on the extensive farming system.

In this paper, we studied the effect of COVID-19 restrictive measures that were imposed on Greece due to the pandemic on the extensive farming system of sheep and goats. Specifically, the objectives were as follows:

a. to describe the breeders' profile and the structural characteristics of their holdings,
b. to investigate the effect of COVID-19 and the restrictive measures on the parameters of sheep and goat extensive farming systems, and
c. to examine whether the breeders' profile and the structural characteristics of their holdings are related to the parameters of sheep and goat extensive farming systems during the COVID-19 restrictive measures.

## 2 Materials and methods

### 2.1 Study area

The study was conducted in two local communities (Livadi and Farmaki) in the municipality of Elassona, Larissa Prefecture, central Greece, from July to October 2020. The climate of the area is characterized as transitional from the typical Greek Mediterranean to middle European type (Köppen climate classification: Csa) with an annual rainfall of 423.2 mm and average annual temperature of 15.7 ${}^{\circ }C$ . The topography is characterized by mild to steep slopes in places. The bedrock consists mainly of gneisses and limestones in shallow soils, with moderate to intense erosion. The pasture types that have been recorded in the area are grasslands, shrublands, and woodlands (Damalis, 2021).

### 2.2 Husbandry system

This geographical area was selected as it is considered one of the country's largest and most important livestock husbandry centers due to its long history of extensive sheep and goat farming (Kaimakamis, 2017). Sheep and goats are exploiting communally the pastures, which provide feed to the animals for 9–10 months in a year (Damalis, 2021). Alternative forage resources are used for the rest of the year period, such as temporary pastures of annual winter cereals (mainly barley and wheat) in early spring and cereal stubble fields after crop harvesting during the summer. Additionally, the majority of breeders (96 %) feed sheep and goats with high quantities (on average 2.14 and 1.77 $\mathrm{kg}\;{\mathrm{animal}}^{-1}\;d^{-1}$ , respectively) of purchased feedstuffs (concentrates and roughages) to increase milk production (Damalis, 2021).

### 2.3 Measurements

The study was based on the collection of primary data through a semi-structured questionnaire that was completed during a face-to-face personal in-depth interview with small ruminant breeders in the area ( N=94 ), who considered livestock husbandry to be their main occupation and did not have any other economic activities except for extensive sheep and goat farming. These breeders were selected from the national database of OPEKEPE (Payment and Control Agency for Guidance and Guarantee Community Aid) where all breeders annually submit their application for income support via the Integrated Administration and Control System (IACS). This is the most accurate database of the country as it provides information about the animal capital and economic activities of breeders. The breeders were informed about the study in person, by telephone, and/or by email, and then the interviews were arranged. All the breeders responded to the call.

Firstly, we prepared a preliminary questionnaire that was addressed to six known small ruminant breeders, with whom we had previously collaborated. This was done to ensure that the questions were clear to the breeders, to identify any new questions that should be included in the final questionnaire, and to evaluate the flow of the interview. Following this, we finalized the content and order of the questions to achieve the objectives of the study. The questionnaire was prepared in Greek, and then it was translated into English for the purposes of the present paper. It is available as a Supplement (Sect. S1). The questionnaire was made up of 32 questions organized in four sections. The first involved questions about the national farm reference number, the breeder's name, and the local community where the farm is located. In the second section, there were questions on the breeder's profile (gender, age group, level of education, and other activities in addition to livestock husbandry), and in the third part, there were questions about the characteristics of the holding, such as the type of holding (individual or family), the number of family employees, the number of non-family employees, the applied farming system (sedentary or transhumant), the kind and number of raised livestock, the structure of the flock, and the orientation of production (milk, meat, or mixed). The fourth section was structured in four subsections and had questions regarding the effect of the COVID-19 disease and the restrictive measures imposed in the country due to the pandemic on the following:

1. breeders' health and their daily work routine,
2. animal management practices (daily movement of animals in pastures, quantity of the administered feedstuffs, animal capital size, and the applied hygienic conditions in the holdings),
3. animal product retail (demand and market prices of milk and meat), and
4. labor demand concerning non-family employees and lack of laborers and/or workers.

The interviews followed the semi-structured questionnaire and were accompanied by a script that was developed to guide the interview process and assist the interviewer in completing the questionnaire as well as to mitigate any possible concerns of the breeders about the purpose of the study, their consent, and the confidentiality of their participation and responses. Each interview began with a short introduction by the interviewer as a researcher collecting data for a project concerning the effects of restrictive measures that were imposed in the country due to the COVID-19 pandemic on the extensive farming system of sheep and goats. Then the interviewer informed the breeders that the collected data would be used only for scientific purposes and that the study had not received any grants as well as assuring them that all the provided information will be kept confidential, and the interviewee's name will not appear anywhere. After that, the interviewer asked the questions to the breeders and completed the questionnaires. For one question (no. 12) it was necessary to provide a definition about the meaning of some terms to the breeders (e.g., sedentary and transhumant) because the majority of the breeders were not familiar with these terms and had not heard them before. In addition, the interviewer provided more details on the following three questions. (1) Question no. 18 is as follows: “If yes, how were you affected?” The answer “directly” referred to their infection by the COVID-19 disease and “indirectly” to the psychological effect due to increased stress and anxiety. (2) Question no. 21 is as follows: “Has there been any change in the daily movement of animals in pastures?” Daily movement referred to the duration of grazing time in pastures as well as the daily sheep and goat travel distance to cover their nutritional needs. (3) Question no. 24 is as follows: “Has there been any improvement in the hygienic conditions of your holding?” The hygienic conditions referred to keeping the farm environment cleaner, wearing a mask when coming in contact with the workers, washing hands more often, and using antiseptics. At the end, the interviewer asked the breeders if they had any questions or any comments and informed them that there may be subsequent contact if there is a need to clarify information or ask additional questions. The interviewer also provided contact information (address, mobile number, and email address) and thanked the breeders for participating in the study.

One interviewer performed all the interviews in the same place (in a private office). The interviews were not recorded because of concerns that would have made the breeders reluctant to participate in the study. There were no questions or comments by the breeders and no additional information was requested by the interviewer. The duration of each interview was about 25 min . The interviews were conducted from 12 August to 14 September 2020.

Collecting primary data in this way is advantageous because there are people who cannot write their answer or do not write as frequently as they speak. People who usually tend to ignore a questionnaire may be willing to talk to someone who is interested in what they have to say about their work and their problems (Harris and Brown, 2010; Jacob and Furgerson, 2012).

### 2.4 Statistical analysis

Descriptive statistics (frequency and valid percentage) were used to analyze and present the data (Zar, 2010). All variables that were used in the study are listed below.

1. Variables of the breeders' profile: “gender” with two levels (0 = female, 1 = male), “age group” with five levels (0 = 21–30 age group, 1 = 31–40 age group, 2 = 41–50 age group, 3 = 51–60 age group, 4 = 61–70 age group), and “educational level” with five levels (1 = elementary, 2 = middle school, 3 = high school, 4 = technological educational institute degree, 5 = Bachelor degree).
2. Variables of characteristics of the holdings: “type of holding” with two levels (1 = individual, 2 = family), “number of family employees” with three levels (1 = 1 family employee, 2 = 2 family employees, 3 = 3 family employees), “number of non-family employees” with three levels (0 = 0 non-family employee, 1 = 1 non-family employee, 2 = 2 non-family employees), “farming system” with two levels (1 = sedentary, 2 = transhumant), “kind of raised animal species with two levels (1 = sheep, 2 = goats), “structure of flock in the holding” with three levels (1 = sheep, 2 = goats, 3 = mixed), and “orientation of production” with three levels (1 = milk, 2 = meat, 3 = mixed).
3. Variables of sheep and goat extensive farming system during the COVID-19 restrictive measures: “breeders' health” with three levels (0 = not infected, 1 = indirectly infected, 2 = directly infected), “breeders' daily work routine” with three levels (0 = neutrally, 1 = negatively, 2 = positively), “daily movement of animals in pastures” with three levels (0 = no change, 1 = increased, 2 = reduced), “quantity of the administered feedstuffs” with three levels (0 = no change, 1 = increased, 2 = reduced), “animal capital size” with three levels (0 = no change, 1 = increased, 2 = reduced), “hygienic conditions in the holding” with two levels (0 = no change, 1 = improved), “demand for animal products” with three levels (0 = no change, 1 = increased, 2 = reduced), “price of animal products” with three levels (0 = no change, 1 = increased, 2 = reduced), “number of non-family employees” with three levels (0 = no change, 1 = increased, 2 = reduced), and “lack of new laborers/workers” with two levels (0 = no, 1 = yes).
4. Quantitative variables: “number of raised sheep” ranged from 0–730 and “number of raised goats” ranged from 0–320.

The variables of the breeders' profile and the characteristics of the holdings were considered to be independent variables, while the variables of sheep and goat extensive farming systems during the COVID-19 restrictive measures were considered dependent ones. Logistic regression modeling was used to test the relation between dependent variables and independent variables, which is a model procedure suitable for estimating the probability of a discrete, nominal, or ordinal outcome given an input scale and/or categorical variables (Hosmer et al., 2013). In the present study, the variables were either binary or nominal with more than two levels. If needed, a reduction in the levels of the dependent and independent variables was applied for achieving a better performance of the modeling procedure. Specifically, the variables for the breeders' daily work routine, age group, number of family employees, and number of non-family employees were binarized, while the educational level was reduced to three levels.

Due to the availability of several independent variables, a backward stepwise selection procedure was used for the selection of the best-fit model, as described and suggested by Wang et al. (2007). The variables “demand for animal products” and “price of animal products” were not modeled, as they were considered exogenous to the breeders' profile and the characteristics of the holding. They were examined only for the effect of the pandemic restrictive measures.

Initially, all the independent variables were entered in the model, and in each step, the non-significant variables were eliminated from the model. The backward selection stopped on the step that the model fits best. The stopping criterion was set to the interval 0.25 ≤ a ≤ 0.35, where a is the significance level, and the log-likelihood ratio was used as the selection criterion (Wang et al., 2007). In addition, the logistic regression assumptions were examined. The quantitative variables were transformed by subtracting the mean from each case, a procedure that helps the logistic regression algorithm to converge (Gray and Kinnear, 2012). Also, no multicollinearity was detected among the independent variables (Hair et al., 2006). The significance level for testing the importance of the variables that were included in the final logistic regression model was set at a = 0.05 ( P≤0.05 ). All statistical and visualization procedures were carried out with the use of SPSS v. 27 software (SPSS, 2017; George and Mallery, 2022).

**Table 1:** Breeders' profile and structural characteristics of sheep and goat holdings in the study area.

				Frequency	Valid percent (%)
Breeders' profile	Gender	Female		26	27.7
		Male		68	72.3
	Age group	21–30		5	5.3
		31–40		22	23.4
		41–50		33	35.1
		51–60		20	21.3
		61–70		14	14.9
	Educational level	Elementary		40	42.6
		Middle school		34	36.2
		High school		14	14.9
		Technological educationalinstitute degree		5	5.3
		Bachelor degree		1	1.0
Structural characteristics of the holdings	Type of holding	Individual holding		7	7.4
		Family holding		87	92.6
	Number of employees	Family employees	1	23	24.5
			2	66	70.2
			3	5	5.3
		Non-family employees	0	61	64.9
			1	29	30.9
			2	4	4.2
	Farming system	Sedentary		92	97.9
		Transhumant		2	2.1
	Kind of raised livestock	Sheep		59	62.8
		Goats		7	7.5
		Mixed		28	29.7
	Orientation of production	Milk		1	1.0
		Meat		6	6.4
		Mixed		87	92.6

## 3 Results

### 3.1 Breeders' profile and structural characteristics of the holdings

The majority of the breeders were men (Table 1) with ages up to 50 years old (63.8 %). Regarding the educational level of breeders, 51.1 % were middle and high school graduates, and less than half were elementary school graduates, while 6.3 % had a degree from a technological educational institute and a Bachelor degree.

The livestock holdings were mainly family type, while the non-family enterprises were at a lower percentage. In the holdings, there were one to three members of the breeder's family employed (Table 1). Most of the holdings employed two members of the breeder's family, while only 35.1 % of the holdings employed one to two laborers or workers that were non-family members of the breeder. The vast majority of the breeders were practicing extensive farming systems, with a very small percentage practicing transhumance. Sheep were the dominant raised livestock species followed by goats, while there were no holdings with cattle. Sheep and goat flocks were pure or mixed with the greater percentage being pure sheep flocks. Regarding the orientation of production, the majority of farms were mixed, and very few of them produced meat, while a small minority produced milk.

**Table 2:** Percentage (%) of holdings according to the number of raised sheep and goats.

Number of	Percentage (%) of small			
raised animals	ruminant holdings			
	Sheep	Total	Goats	Total
1–49	1.23		51.03	51.03
50–99	0.36		8.29	
100–149	2.12		10.34	
150–199	7.05		11.03	
200–249	14.99	25.75	10.34	40.00
250–299	15.86		4.14	
300–349	18.52	34.38	4.83	8.97
350–399	12.70		-	-
400–449	19.05		–	–
450–499	3.53		–	–
500–549	1.94		–	–
> 550	2.65	39.87	–	–
Total		100.00		100.00

A total of 24 961 sheep and 3220 goats were raised in the area with holdings having on average ( M ± SE) 265.5 ± 13.2 sheep and 34.3 ± 6.9 goats. The majority of breeders (60.13 % and 51.03 %) were rearing from 1 to 349 sheep and from 1 to 49 goats (Table 2), while there were no farms with a capacity of more than 320 goats.

**Figure 1:**
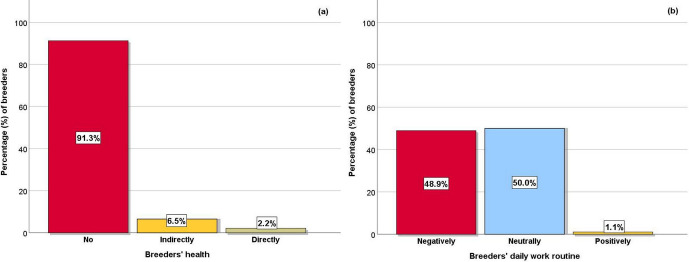
**(a)** Effect of the COVID-19 pandemic on breeders' health and **(b)** effect of the restrictive measures due to the pandemic on breeders' daily work routine.

### 3.2 Effect of restrictive measures on the parameters of the extensive farming system

The majority of the breeders was not infected by COVID-19 (Fig. 1a). However, the restrictive measures mostly affected the health of breeders indirectly (psychologically), having a very small direct effect on them. Half of the breeders responded that the COVID-19 restrictive measures had a neutral effect on their daily work routine, while a little less than half of them responded that the measures had a negative impact, and only one respondent thought that the measures had a positive effect on their work (Fig. 1b).

**Figure 2:**
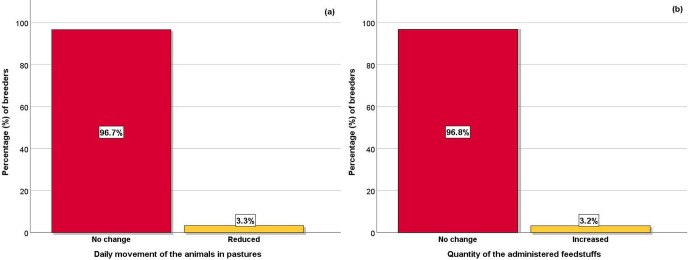
Effect of COVID-19 restrictive measures on **(a)** small ruminant daily movements in pastures and **(b)** the quantity of the administered feedstuffs.

The majority of the breeders did not reduce the daily movements of their animals in pastures during the COVID-19 restrictive measures (Fig. 2a). However, a few of them responded that they reduced the daily movements of animals in pastures. In addition, they did not change the quantity of administered feedstuffs to the animals (Fig. 2b), although there was a small percentage of breeders that increased the quantity of feedstuffs to their animals.

**Figure 3:**
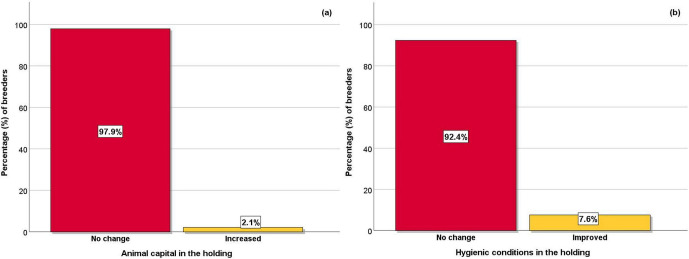
Effect of COVID-19 restrictive measures on **(a)** animal capital and **(b)** the hygienic conditions in the holdings.

In terms of the rearing of sheep and goats, in the majority of the holdings, there was no change in the size of animal capital, while in a small minority there was an increase (Fig. 3a). In addition, the majority of breeders responded that they did not change the hygienic conditions in their livestock facilities (Fig. 3b) in contrast to a small minority who improved them.

**Figure 4:**
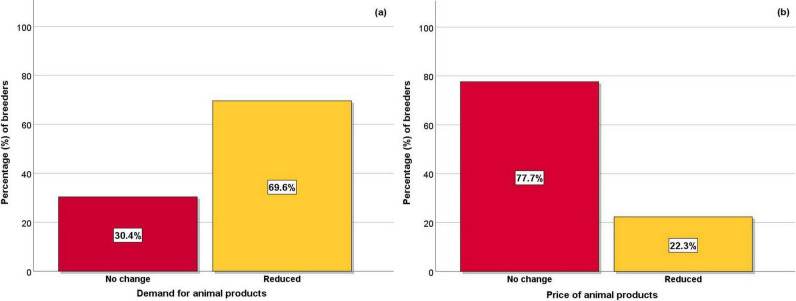
Percentage (%) of small ruminant breeders who observed a reduction in the **(a)** demand for animal products and the **(b)** price of animal products during the COVID-19 restrictive measures.

Furthermore, most breeders responded that they observed a decrease in the demand for produced animal products, while some of them did not observe any change (Fig. 4a). In addition, at a bit less than a quarter of the breeders responded that there was a decrease in the price of animal products during the COVID-19 restrictive measures (Fig. 4b), especially during the period of Easter.

**Figure 5:**
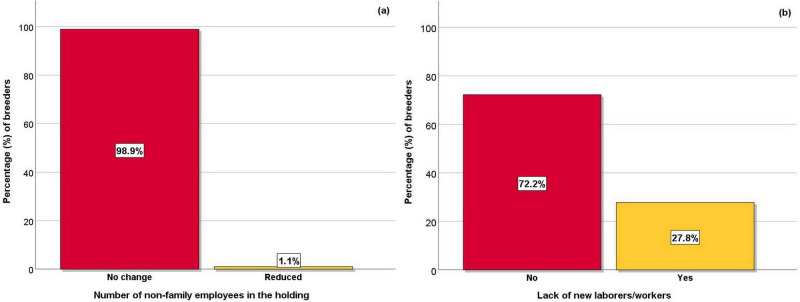
Effect of COVID-19 restrictive measures on the **(a)** number of non-family employees and **(b)** lack of laborers and/or workers.

In the vast majority of the livestock holdings, the number of non-family employees did not change, while in a small minority there was a decrease of two employees (Fig. 5a). In addition, in a bit more than a quarter of the holdings, there was an observed lack of laborers and/or workers due to the COVID-19 restrictive measures imposed to control the pandemic outbreak (Fig. 5b).

**Table 3:** Logistic regression results (sixth step) for the effects of the structural characteristics of the holdings on the breeders' daily work routine.

Step ${}^{*}$	Name of the variable	B ${}^{1}$	SE ${}^{2}$	Wald ${}^{3}$	DF ${}^{4}$	Sig. level ${}^{5}$	Exp(B) ${}^{6}$
Sixth	Type of holding	- 1.943	1.119	3.016	1	0.082	0.143
	Number of raised sheep	- 0.005	0.002	4.339	1	0.037	0.995
	Farming system	- 11.624	191.00	0.004	1	0.951	0.000
	Constant	15.359	191.01	0.006	1	0.936	4.6 × 10 ${}^{6}$

${}^{1}$ B is the regression coefficient, ${}^{2}$ SE is the standard error of B , ${}^{3}$ the Wald statistic ascertains whether a variable is a significant predictor of the outcome, ${}^{4}$ DF is the degrees of freedom of the Wald statistic, ${}^{5}$ Sig. level is the significance level of the Wald statistic, and ${}^{6}$ Exp(B) represents the odds ratio. ${}^{*}$ Variables entered at step 1: age (0 = 21–40 age group, 1 = > 40 age group), educational level (0 = elementary, 1 = middle school, 2 = higher education), family employees (0 = 1 family employee, 1 = 2–3 family employees), non-family employees (0 = 0 non-family employee, 1 = 1–2 non-family employees), type of holding (1 = individual holding, 2 = family holding), sheep number (0–730 heads), orientation of production (1 = dairy, 2 = meat, 3 = mixed), farming system (1 = sedentary, 2 = transhumant).

### 3.3 Relation of the socio-structural characteristics of the holdings with the parameters of the extensive farming system

The breeders' daily work routine was the only dependent variable that was modeled. The backward stepwise selection procedure stopped in step 6, and it included three independent variables: the “type of holding”, the “number of raised sheep”, and the “farming system” (Table 3). Thus, the final logistic regression model was expressed via the following equation: logit=-1.943×(type  of  the  holding)-0.005×(number  of  raised  sheep)-11.624×(farming  system)+15.359 and had a significant fit of the data ( - 2 log-likelihood statistic = 108.522, $\chi^{2}$ = 11.983, P = 0.007). The number of raised sheep was the only significant variable (Wald(1) = 4.339, P = 0.037) in the model. As indicated by the regression coefficient ( B ± SE), when the number of raised sheep is increased by one unit, the natural log⁡ of the odds in favor of the “neutral” answer is decreased by 0.005 ± 0.002 units. Additionally, when the number of raised sheep increases by one unit, the likelihood of the COVID-19 restrictive measures having a neutral effect on the breeders' daily work routine decreases by 0.995, as revealed by the Exp(B) statistic. No other independent variables were included in the model. All the above mean that the probability of a negative effect of COVID-19 restrictive measures on the breeders' daily work routine is increased as the number of raised sheep increases. Thus, the variable “number of raised sheep” makes both a significant and substantial contribution to the regression, while the other two variables do not.

## 4 Discussion

### 4.1 Breeders' profile and structural characteristics of the holdings

In the current study, the majority of small ruminant breeders were male, with one-third of them being female. It seems that the hard working conditions concerning the management and supervision of flocks on farms and pastures do not prevent women from having livestock breeding as their main occupation, thus overcoming gender inequalities in rural areas. The latter has been identified as an important aspect of the European Union policy as the engagement of women in farming provides them with income and financial independence, and it helps maintain vibrant rural areas and communities (Bock, 2015; Shortall, 2015). Our results are in accordance with the results from the study of Tsiaousi and Partalidou (2020), who stated that almost 30 % of farmers in Greece are women. However, Lianou and Fthenakis (2021), in a study concerning the socio-demographic characteristics of dairy sheep and goat farmers, provided contradictory results, highlighting the need for further countrywide investigation.

Moreover, the breeders were of middle age and were educated at the basic educational level. Specifically, the majority of breeders (56.4 %) were 40–60 years old, and only 6.4 % of them had a university degree. This is understandable, as younger generations with a higher level of education might not be interested in selecting sheep and goat breeding as a professional activity, probably due to the hard working conditions and the negative social status that characterizes the profession (Yiakoulaki and Hasanagas, 2019). Regarding the effect of the pandemic and the restrictive measures on the Greek population, it has been found that older age groups and females were considered more vulnerable to psychological disorders (Parlapani et al., 2020).

Small ruminant farming in our study was practiced within the breeder's family, as 92.6 % of the holdings were family type. For this reason, the employees in the holdings were mainly members of the breeder's family (64.9 %), and usually (70.2 %) two members were employed. This finding is in agreement with Sreekumar and Sreenivasaiah (2015), who mentioned that sheep and goat breeding is often considered a family business. The non-family employees are usually hired as seasonal laborers and/or workers to fill in and account for the demands during busy periods (animal reproduction and births). According to data from the Hellenic Statistical Authority (2018), breeders and their family members constitute 40.5 % of the total workforce in livestock holdings and seasonal laborers or workers constitute about 30.9 %.

Extensive breeding is the prevalent system in the study area as it is often the only professional way in semi-mountainous and mountainous areas (de Rancourt et al., 2006). This system is practiced in several areas of the country and pastures located at various altitudinal zones (Yiakoulaki et al., 2003; Evagelou et al., 2008; Yiakoulaki and Papanastasis, 2014; Koidou et al., 2019). In contrast, the transhumant farming system is practiced only by 2.1 % of sheep and goat breeders, confirming the significant decline of this system over time (Yiakoulaki and Papanastasis, 2014).

In the study area, small-scale breeders manage livestock husbandry. In this regard, Hadjigeorgiou (2011) mentioned that the average size of sheep and goat flocks in the whole country was 68.1 and 38.5 animals, respectively, with a great regional differentiation in the mean flock size. In our study, 39.9 % of sheep holdings had bigger flocks (from 350 to > 550 animals), while there were no goat holdings with such a big flock size. Our results are in accordance with the annual census of agriculture and livestock (Eurostat Archive, 2013), which refers to a decrease in the number of the livestock holdings and an increase in their size. Additionally, the majority of holdings (92.6 %) were oriented towards mixed production (milk and meat ). In this regard, de Rancourt et al. (2006) mentioned that breeders often combine the higher income from dairy products with the higher subsidies from the European Union awarded for the production of meat.

### 4.2 Effect of restrictive measures on the parameters of sheep and goat extensive farming systems

COVID-19 did not affect breeders' health as very few of them were infected by the virus. However, the pandemic and the restrictive measures affected the mental health of a small number of breeders, who responded that they had increased stress or anxiety. This is understandable due to the uncertainty regarding their health and future as well as the pandemic's economic, political, and social consequences (Hashem et al., 2020; Parlapani et al., 2020; Reile et al., 2021).

In addition, 49 % of the breeders responded that their daily work routine had been negatively impacted by the pandemic and the restrictive measures for more than a month. That restriction obviously occurred to decrease the possibility of a COVID-19 infection during their contact with workers or animal feed suppliers and other suppliers as well (Taylor et al., 2020). Moreover, it is worth mentioning that a very small minority of breeders (1.1 %) considered the pandemic to be a positive factor for their professional activities. That could be due to the potential emergence of new perspectives in their future professional activities (e.g., selling their products to individual households online).

The extensive farming system is based on livestock movements in pastures for the exploitation of natural forage resources through grazing. Small ruminant breeders lead their animals to pastures every morning and return them to stables at night (Papanastasis, 2009; Yiakoulaki and Papanastasis, 2014). Grazing time in pastures ranges from 5–10 $h\;d^{-1}$ and depends on the kind of animals, the season of grazing, the pasture altitudinal zone, and the availability and nutritional value of forage (Zarovali et al., 2006; Evangelou et al., 2008). Furthermore, it is mentioned by Evangelou et al. (2014) that sheep and goats travel long distances daily (7.5 and 9.0 km , respectively) to cover their nutritional needs. However, in the study area, the mean daily sheep and goat travel distance was shorter (4.9 $\mathrm{km}\;d^{-1}$ ) according to Damalis (2021). The vast majority (96.7 %) of breeders did not differentiate the daily animal movements in pastures or the quantity of the administered feedstuffs. On the contrary, according to Hashem et al. (2020), the COVID-19 restrictive measures resulted in restrictions on animal movements to the pastures and shortages of available feedstuffs. Along the same line of thought, Zhang (2020) referred to difficulties in accessing animal feedstuffs and restrictions in the continuous feeding of farmed animals due to social distancing measures and restrictions on the import–export of goods. However, the abovementioned obstacles address intensive livestock production, underlining the fragility of this system (Marchant-Forde and Boyle, 2020), while in our study the breeders did not seem to face such obstacles.

It could be expected that the pandemic and the restrictive measures would improve the hygienic conditions in the farms; however, the majority of breeders did not seem to improve the cleanliness conditions in their farms during the first wave of the pandemic. Only 7.4 % of breeders followed the new strict hygiene rules in the farms in the context of public health protection.

The majority of sheep and goat breeders (97.9 %) maintained the animal capital of their holdings during the first wave of the pandemic and the restrictive measures. However, this does not necessarily mean that the pandemic had a positive effect on the sustainability of holdings, as not placing the animals on the market financially burdens the breeders (Marchant-Forde and Boyle, 2020).

According to FAO (2020c), the COVID-19 pandemic and the restrictive measures negatively affected the supply chain and the demand for animal products (milk and meat) on a global level. That could be because of reduced production and supply and/or the pause of sales of these products for leisure activities, tourist businesses, and local markets (Hashem et al., 2020; Ijaz et al., 2021; Brumă et al., 2021). In the current study, the majority of the breeders (67 %) responded that there was a decrease in the demand for their animal products during the pandemic restrictive measures. Specifically, they mentioned that there was a 10 %–30 % decrease in the demand for milk and a 20 %–40 % decrease in the demand for meat. This is probably attributed to increasing unemployment (FAO, 2020a) and changes in consumer dietary habits. The changes in the consumption of animal products occurred due to reduced purchasing power, changes in priorities, and concerns and doubts about the safety of animal products (Hashem et al., 2020). Montanari et al. (2021) reported low consumer demand for sheep and goat products in a study requested by the European Parliament's Committee on Agriculture concerning the quantitative and qualitative analysis of the impact of COVID-19 on European agriculture. In our study, about 22.3 % of the breeders reduced the prices of their products, especially in the Easter period (April), due to the decreased demand. This finding is confirmed by the Consumer Price Index (Hellenic Statistical Authority, 2020), according to which the price of goat and sheep meat decreased by 3.5 % in April compared to March, while the price of milk decreased by 2 %. During the celebration of Easter, there is increased sheep and goat meat consumption due to the Greek cultural and nutritional habits. However, the restrictions imposed in the country to control the spread of the pandemic contributed to a decrease in meat prices because of the low meat consumption by consumers.

Further, the breeders did not reduce the compensation of their non-family employees. Also, in 26.6 % of the holdings, there was an increased need for laborers and workers, but there was no availability. That could have been the case due to the general restrictions on citizen movements and consequently of workers in the livestock enterprises or the possibility of the workers being infected by COVID-19 and their isolation, which in turn affected the availability of the workforce (Hashem et al., 2020).

### 4.3 Socio-structural characteristics of the holdings and parameters of the extensive farming system

The COVID-19 restrictive measures appear to be independent of breeders' profile. This is understandable as the restrictive measures were for all breeders regardless of their gender, age group, and educational level. Breeders' daily work routine was the only dependent variable that created diversity in the breeders' answers about the effect of the COVID-19 restrictive measures. Contrary to the “no change” answer of the other dependent variables, almost half of the respondents said that these measures had a negative effect on their daily work routine, while the other half evaluated this effect as neutral. The applied logistic regression model showed that the number of sheep in the holdings was related to this effect and that it is more likely that in holdings with a high number of sheep, the restrictive measures had a negative effect on the breeders' daily work routine. This is understandable due to the increased needs for nutrition, milking, and housing of big sheep flocks in the farms as well as their handling in pastures. The calls to stay at home, the lockdown, the social distancing, and the limitations in breeders' daily mobility to and from work for taking care of their animals and moving the live animals and animal products to the markets affected human-dependent services in the farms. Moreover, the shortage of workers due to the lockdown, quarantine, travel bans, and border control affected the daily work routine in the farms. In our study 28 % of the breeders were facing a labor shortage. Thus, it is obvious that the negative effect of restrictive measures on breeders' daily work routine is more pronounced in holdings with large flocks of sheep. Similarly, Brzáková et al. (2021) reported that various large farms in the Czech Republic encountered labor shortages during the COVID-19 crisis, which mainly affected the milking and feeding of animals in the farms.

## 5 Conclusions

In conclusion, the strict restrictive measures that were imposed to control the spreading of the first wave of the COVID-19 pandemic during March–May 2020 do not seem to have significantly impacted the practicing of extensive sheep and goat farming in the area of Elassona, central Greece. Only the breeders' daily work routine was significantly affected by the COVID-19 restrictive measures. Particularly, holdings with a high number of sheep were more likely to experience negative effects. On the other hand, the majority of breeders continued their activities without changing the movements of their animals in pastures, the amount of the given feedstuffs, the hygienic conditions in farm installations, or the compensation to their employees. Further, a decrease in the demand for produced animal products (meat and milk) and a decrease in their prices were also observed.

## Supplement

10.5194/aab-65-157-2022-supplement The supplement related to this article is available online at: https://doi.org/10.5194/aab-65-157-2022-supplement.

## Data Availability

The original data are available upon request from the corresponding author.
