# Supplementary material for: The effects of restrictive measures due to the COVID-19 pandemic on the extensive farming system of small ruminants
Source: Arch Anim Breed. 2022 May 2;65(2):157–69. doi: 10.5194/aab-65-157-2022 (PMC9069180; doi:10.5194/aab-65-157-2022)
Supplement: The supplement related to this article is available online at: https://doi.org/10.5194/aab-65-157-2022-supplement. [file aab-65-157-supplement.pdf]

Supplement of Arch. Anim. Breed., 65, 157–169, 2022
https://doi.org/10.5194/aab-65-157-2022-supplement
© Author(s) 2022. CC BY 4.0 License.

Open Access
Archives Animal Breeding

Supplement of

The effects of restrictive measures due to the COVID-19 pandemic on the extensive farming system of small ruminants

Maria D. Yiakoulaki et al.

Correspondence to: Maria D. Yiakoulaki (yiak@for.auth.gr)

The copyright of individual parts of the supplement might differ from the article licence.

Supplement (Sect. S1): A questionnaire for the effects of restrictive measures due to Covid-19 pandemic on the extensive farming system of sheep and goats.

Consent Provision		
**Please provide your consent to participate in this study**	☐ Yes, I consent	☐ No, I do not consent
**Research's Code Number:**		
**Date:**		

No	Question	
**1**	**Livestock holding's info**	
**01**	**Farm's National Reference Number (No):**	
**02**	**Breeder's Name:**	
**03**	**In which Local Community is your farm located?**	

2	Breeder's profile			
**04**	**Gender**	Male ☐		Female ☐
**05**	**Please indicate your age group**	21-30 years ☐, 31-40 years ☐, 41-50 years ☐, 51-60 years ☐, 61-70 years ☐		
**06**	**Please indicate your educational level**	Primary School ☐	Middle School ☐	High School ☐
		Technological Educational Institute's degree ☐		Bachelor's degree ☐
**07**	**Do you have any other activities in addition to animal husbandry?**	No ☐		Yes ☐
**08**	**If yes, please specify.**			

3	Livestock holdings characteristics			
**09**	**Please define the type of your holding**	Individual ☐		Family ☐
**10**	**Number of family employees in your holding (No)**	1 ☐ 2 ☐ 3 ☐		
**11**	**Number of non-family employees in your holding (No)**	0 ☐ 1 ☐ 2 ☐		
**12**	**Please define the applied farming system in your holding**	Sedentary ☐<br>(Animals can freely utilize the pastures that are allocated to the village where the breeder resides)		Transhumant ☐<br>(Seasonal movement of animals between winter and summer pastures)
**13**	**Kind of raised animal species**	Sheep ☐		Goats ☐
**14**	**Number of animal species (No)**			
**15**	**Please specify the structure of flock in your holding**	Sheep ☐	Goats ☐	Mixed ☐
**16**	**Please specify the orientation of production in your holding**	Milk ☐	Meat ☐	Mixed ☐

**4**	**Effect of Covid-19 and Restrictive Measures on:**			
**4.1**	**Breeders**			
**17**	**Did Covid-19 pandemic affect your health?**	No ☐	Yes ☐	
**18**	**If yes, how were you affected?**	Directly ☐ (Infected)	Indirectly ☐ (Psychologically, due to the increased stress and anxiety)	
**19**	**How did the restrictive measures affect your daily work routine?**	Negatively ☐	Neutrally ☐	Positively ☐
**20**	**Please specify for how long your daily work routine was affected**			
**4.2**	**Animal Management Practices**			
**21**	**Has there been any change in the daily movement of animals in pastures?** (In the meaning of duration of grazing time in pastures as well as the daily sheep and goats travel distance to cover their nutritional needs)	No change ☐	Increased ☐	Reduced ☐
**22**	**Has there been any change in the quantity of the administered feedstuffs?**	No change ☐	Increased ☐	Reduced ☐
**23**	**Has there been any change in the animal capital of your holding?**	No change ☐	Increased ☐	Reduced ☐
**24**	**Has there been any improvement in the hygienic conditions of your holding?** (In the meaning of keeping the farm environment cleaner, wearing a mask when coming in contact with the workers, washing hands more often, and using antiseptics)	No change ☐	Yes, I improved them ☐	
**4.3**	**Animal Products**			
**25**	**Has there been any change in the demand for animal products?**	No change ☐	Increased ☐	Reduced ☐
**26**	**Which animal products were affected?**	Milk ☐	Meat ☐	
**27**	**How much was approximately the change (%)?**	10 ☐ 20 ☐ 30 ☐ 40 ☐ More ☐	10 ☐ 20 ☐ 30 ☐ 40 ☐ More ☐	
**28**	**Has there been any change in the price of animal products?**	No change ☐	Increased ☐	Reduced ☐
**29**	**If yes, which products' price was affected?**	Milk ☐	Meat ☐	
**30**	**Which time period did you observe the change?**			
**4.4**	**Labor-Personnel**			
**31**	**Has there been any change in the number of non-family employees of your holding during the restrictive measures?**	No change ☐	Increased ☐	Reduced ☐
**32**	**Was there an observed lack of new laborers/workers during the restrictive measures?**	No ☐	Yes ☐	
	**Do you have any questions? Are there any comments?**			
